# Dicarbon­yl(pyrazine-1,3-dithiol­ato-κ^2^ *S*,*S*′)bis­(trimethyl­phosphane-κ*P*)iron(II)

**DOI:** 10.1107/S1600536811048574

**Published:** 2011-11-19

**Authors:** Shang Gao, Qian Duan, Chun-ai An, Da-yong Jiang

**Affiliations:** a School of Materials Science and Engineering, Changchun University of Science and Technology, No. 7989 Weixing Road, Changchun 130022, People’s Republic of China

## Abstract

The title compound, [Fe(C_4_H_2_N_2_S_2_)(C_3_H_9_P)_2_(CO)_2_], was obtained as a mononuclear by-product during the treatment of [Fe_2_(μ-S_2_C_4_N_2_H_2_)(CO)_6_] in excess trimethyl­phosphane. The Fe atom is six-coordinated by two thiol­ate S atoms, two phosphane P atoms and two carbonyl C atoms in a distorted octa­hedral geometry. The average Fe—C(O) distance (1.771 Å) is relatively shorter than that of its parent hexa­carbonyl­diiron compound, and differs by 0.511 Å from the average Fe—P(Me)_3_ distance. The five-membered FeC_2_S_2_ chelate ring plane is close to being perpendicular to the P/Fe/P plane [86.5 (2)°].

## Related literature

For general background to iron sulfides, see: Cody *et al.* (2000 ▶); Georgakaki *et al.* (2003 ▶); Capon *et al.* (2005 ▶); Song (2005 ▶); Li *et al.* (2005 ▶); Liu & Xiao (2011 ▶). For related structures and the synthesis, see: Durgaprasad *et al.* (2011 ▶).
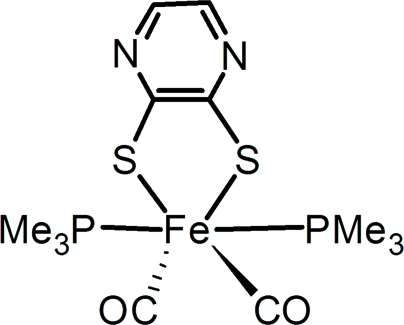


## Experimental

### 

#### Crystal data

[Fe(C_4_H_2_N_2_S_2_)(C_3_H_9_P)_2_(CO)_2_]
*M* *_r_* = 406.21
Orthorhombic, 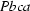

*a* = 12.2078 (10) Å
*b* = 11.951 (1) Å
*c* = 25.326 (2) Å
*V* = 3694.9 (5) Å^3^
*Z* = 8
Mo *K*α radiation
μ = 1.22 mm^−1^
*T* = 273 K
0.30 × 0.25 × 0.20 mm

#### Data collection

Bruker APEXII CCD area-detector diffractometer
Absorption correction: multi-scan (*SADABS*; Bruker, 1997 ▶) *T*_min_ = 0.711, *T*_max_ = 0.793
18679 measured reflections
3628 independent reflections
3166 reflections with *I* > 2σ(*I*)
*R*_int_ = 0.025

#### Refinement

*R*[*F* ^2^ > 2σ(*F* ^2^)] = 0.029
*wR*(*F* ^2^) = 0.073
*S* = 1.09
3628 reflections
190 parameters
H-atom parameters constrained
Δρ_max_ = 0.30 e Å^−3^
Δρ_min_ = −0.56 e Å^−3^

### 

Data collection: *APEX2* (Bruker, 2005 ▶); cell refinement: *SAINT-Plus* (Bruker, 2001 ▶); data reduction: *SAINT-Plus*; program(s) used to solve structure: *SHELXS97* (Sheldrick, 2008 ▶); program(s) used to refine structure: *SHELXL97* (Sheldrick, 2008 ▶); molecular graphics: *SHELXTL* (Sheldrick, 2008 ▶); software used to prepare material for publication: *SHELXTL*.

## Supplementary Material

Crystal structure: contains datablock(s) global, I. DOI: 10.1107/S1600536811048574/kp2368sup1.cif

Structure factors: contains datablock(s) I. DOI: 10.1107/S1600536811048574/kp2368Isup2.hkl

Additional supplementary materials:  crystallographic information; 3D view; checkCIF report

## Figures and Tables

**Table 1:** Selected bond lengths (Å)

Fe1—C8	1.761 (2)
Fe1—C7	1.780 (2)
Fe1—P1	2.2793 (6)
Fe1—P2	2.2840 (6)
Fe1—S2	2.3058 (6)
Fe1—S1	2.3170 (6)

**Fig. 1.**
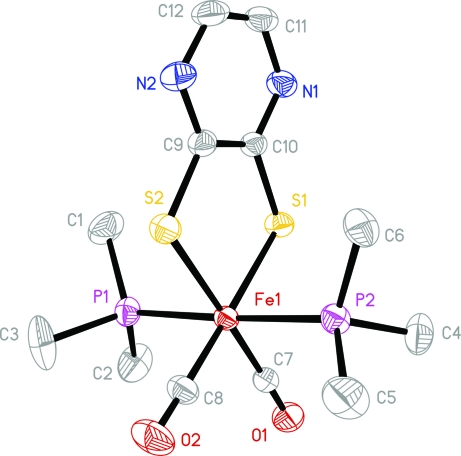
The molecular structure of the title compound with displacement ellipsoids drawn at 30% probability level.

## supplementary crystallographic information

### Comment

Recently iron sulfides have been proposed as being central to the emergence of life due to their structural resemblance to the active site of hydrogenases (Cody *et al.*, 2000, Georgakaki *et al.*, 2003, Capon *et al.*, 2005). Various dinuclear complexes featured [Fe_2_(µ-S*R*)_2_(CO)_6-__γ_L_γ_] (*L* = CO, P*R*_3_*et al.*, γ = 1 or 2) have been investigated as the structural and functional models for the active site of [FeFe]-hydrogenases (Song, 2005, Li *et al.*, 2005, Liu & Xiao, 2011). [Fe_2_(µ-S_2_C_4_N_2_H_2_)(CO)_6_] (Durgaprasad *et al.*, 2011) was prepared for the purpose to lower the reduction potentials of the iron sulfides. When we investigated the CO displacement of above complex by PMe_3_, a mononuclear byproduct was obtained accompanied with PMe_3_-disubstituted diiron compounds. Herein, we report this crystal structure.

In the title compound the central Fe atom is six-coordinated by the two thiolate-sulfur atoms, two phosphane-phosphorus atoms, and two carbonyl-carbon atoms in a distorted octahedral geometry (Fig. 1 and Table 1). The average Fe—C(O) distance (1.77 Å) is relatively shorter than that of its parent hexacarbonyl diiron compound [Fe_2_(µ-S_2_C_4_N_2_H_2_)(CO)_6_] (Durgaprasad *et al.*, 2011), and differs by 0.51 Å from the average Fe—P(Me)_3_ distance, consistent with the better donating role of the tertiary phosphane ligands *vs.* the carbonyl groups. The two S—Fe bonds are nearly perpendicular, and S1—Fe1—S2 angle is 89.198 (19) °. The P1—Fe1—P2 angle is quasilinear [177.45 (2) °] and the deviation of the iron atom from the calculated plane of the –SC_4_N_2_H_2_S– bridge is 0.126 Å. The angle between the calculated rigid dithiolate bridge and the P1Fe1P2 plane deviates from 90° by 3.2° for the title compound, resulting in the asymmetric molecular structure.

### Experimental

Commercially available materials, Me_3_NO and trimethylphosphane were reagent grade and used as received. The starting material [Fe_2_(µ-S_2_C_4_N_2_H_2_)(CO)_6_] was prepared according to the literature procedure (Durgaprasad *et al.*, 2011). [Fe_2_(µ-S_2_C_4_N_2_H_2_)(CO)_6_] (0.42 g, 1.0 mmol) and degassed CH_3_CN (20 ml) was stirred in an argon-filled Schlenk flask until the salvation was completed. Me_3_NO (0.24 g, 2.2 mmol) was added to the above solution in one portion. The mixture was changed to dark red after 10 min. Then the trimethylphosphane (0.15 g, 2.0 mmol) was added dropwise. The solvent was allowed to evaporate on a rotary evaporator after 20 min. The crude product was purified by column chromatography on Al_2_O_3_, using CH_2_Cl_2_/hexane as eluent, yielded two bands. The coral band was collected and the crystals of the title compound suitable for X-ray study were obtained by the recrystallization in the CH_2_Cl_2_/pentane solution (yield 0.12 g, 30%).

### Refinement

The H atoms attached to C were placed in geometrically calculated positions (C—H = 0.93–0.97 Å) and refined as riding, with *U*_iso_(H) = 1.2*U*_eq_(C).

### Crystal data

**Table:**

[Fe(C_4_H_2_N_2_S_2_)(C_3_H_9_P)_2_(CO)_2_]	*F*(000) = 1680
*M**_r_* = 406.21	*D*_x_ = 1.460 Mg m^−^^3^
Orthorhombic, *P**b**c**a*	Mo *K*α radiation, λ = 0.71073 Å
Hall symbol: -P 2ac 2ab	Cell parameters from 9947 reflections
*a* = 12.2078 (10) Å	θ = 2.3–27.5°
*b* = 11.951 (1) Å	µ = 1.22 mm^−^^1^
*c* = 25.326 (2) Å	*T* = 273 K
*V* = 3694.9 (5) Å^3^	Block, orange
*Z* = 8	0.30 × 0.25 × 0.20 mm

### Data collection

**Table:**

Bruker APEXII CCD area-detector diffractometer	3628 independent reflections
Radiation source: fine-focus sealed tube	3166 reflections with *I* > 2σ(*I*)
graphite	*R*_int_ = 0.025
phi and ω scans	θ_max_ = 26.0°, θ_min_ = 2.3°
Absorption correction: multi-scan (*SADABS*; Bruker, 1997)	*h* = −15→14
*T*_min_ = 0.711, *T*_max_ = 0.793	*k* = −14→8
18679 measured reflections	*l* = −31→31

### Refinement

**Table:**

Refinement on *F*^2^	0 restraints
Least-squares matrix: full	H-atom parameters constrained
*R*[*F*^2^ > 2σ(*F*^2^)] = 0.029	*w* = 1/[σ^2^(*F*_o_^2^) + (0.0363*P*)^2^ + 1.1092*P*] where *P* = (*F*_o_^2^ + 2*F*_c_^2^)/3
*wR*(*F*^2^) = 0.073	(Δ/σ)_max_ = 0.001
*S* = 1.09	Δρ_max_ = 0.30 e Å^−^^3^
3628 reflections	Δρ_min_ = −0.56 e Å^−^^3^
190 parameters	

### Special details

**Table:**

Geometry. All e.s.d.'s (except the e.s.d. in the dihedral angle between two l.s. planes) are estimated using the full covariance matrix. The cell e.s.d.'s are taken into account individually in the estimation of e.s.d.'s in distances, angles and torsion angles; correlations between e.s.d.'s in cell parameters are only used when they are defined by crystal symmetry. An approximate (isotropic) treatment of cell e.s.d.'s is used for estimating e.s.d.'s involving l.s. planes.
Refinement. Refinement of *F*^2^ against ALL reflections. The weighted *R*-factor *wR* and goodness of fit *S* are based on *F*^2^, conventional *R*-factors *R* are based on *F*, with *F* set to zero for negative *F*^2^. The threshold expression of *F*^2^ > σ(*F*^2^) is used only for calculating *R*-factors(gt) *etc*. and is not relevant to the choice of reflections for refinement. *R*-factors based on *F*^2^ are statistically about twice as large as those based on *F*, and *R*- factors based on ALL data will be even larger.

### Fractional atomic coordinates and isotropic or equivalent isotropic displacement parameters (Å^2^)

**Table:**

	*x*	*y*	*z*	*U*_iso_*/*U*_eq_	
Fe1	0.374999 (19)	0.76447 (2)	0.389085 (10)	0.03519 (9)	
S1	0.38945 (4)	0.57419 (4)	0.37296 (2)	0.04420 (13)	
P2	0.39648 (4)	0.71975 (5)	0.47615 (2)	0.04635 (14)	
S2	0.56151 (4)	0.78108 (4)	0.37704 (2)	0.05021 (14)	
P1	0.35858 (4)	0.80265 (5)	0.30127 (2)	0.04756 (14)	
O1	0.13778 (12)	0.74340 (14)	0.39685 (7)	0.0643 (4)	
C7	0.23021 (16)	0.75098 (15)	0.39447 (7)	0.0430 (4)	
C9	0.60519 (15)	0.64370 (18)	0.36700 (7)	0.0446 (4)	
O2	0.38260 (15)	1.00089 (14)	0.41494 (8)	0.0817 (5)	
C10	0.53051 (15)	0.55283 (16)	0.36640 (6)	0.0407 (4)	
N1	0.56545 (15)	0.44788 (15)	0.36180 (6)	0.0519 (4)	
C8	0.37676 (16)	0.90822 (17)	0.40424 (9)	0.0502 (5)	
C12	0.74509 (18)	0.5207 (2)	0.35593 (9)	0.0667 (7)	
H12A	0.8193	0.5059	0.3516	0.080*	
C11	0.6742 (2)	0.4338 (2)	0.35706 (7)	0.0589 (6)	
H11A	0.7018	0.3615	0.3545	0.071*	
C6	0.52289 (18)	0.6505 (2)	0.49459 (9)	0.0626 (6)	
H6A	0.5227	0.6361	0.5319	0.094*	
H6B	0.5839	0.6978	0.4859	0.094*	
H6C	0.5291	0.5811	0.4757	0.094*	
N2	0.71250 (14)	0.62810 (18)	0.36084 (7)	0.0612 (5)	
C2	0.2200 (2)	0.8015 (3)	0.27602 (9)	0.0830 (9)	
H2B	0.2208	0.8183	0.2390	0.125*	
H2C	0.1772	0.8567	0.2943	0.125*	
H2D	0.1883	0.7289	0.2814	0.125*	
C3	0.4080 (3)	0.9401 (3)	0.28305 (11)	0.0997 (11)	
H3A	0.3988	0.9508	0.2457	0.150*	
H3B	0.4842	0.9463	0.2919	0.150*	
H3C	0.3671	0.9961	0.3018	0.150*	
C4	0.29368 (19)	0.6241 (2)	0.50169 (9)	0.0728 (7)	
H4A	0.3080	0.6093	0.5383	0.109*	
H4B	0.2963	0.5552	0.4822	0.109*	
H4C	0.2224	0.6571	0.4981	0.109*	
C1	0.4288 (2)	0.7111 (3)	0.25558 (10)	0.0827 (8)	
H1B	0.4155	0.7358	0.2201	0.124*	
H1C	0.4021	0.6361	0.2598	0.124*	
H1D	0.5060	0.7129	0.2626	0.124*	
C5	0.3912 (3)	0.8351 (3)	0.52247 (11)	0.0983 (11)	
H5A	0.4011	0.8073	0.5577	0.147*	
H5B	0.3213	0.8716	0.5199	0.147*	
H5C	0.4483	0.8876	0.5144	0.147*	

### Atomic displacement parameters (Å^2^)

**Table:**

	*U*^11^	*U*^22^	*U*^33^	*U*^12^	*U*^13^	*U*^23^
Fe1	0.03369 (15)	0.03112 (15)	0.04075 (16)	0.00071 (9)	−0.00012 (10)	0.00066 (10)
S1	0.0391 (2)	0.0349 (2)	0.0586 (3)	−0.00252 (18)	0.00205 (19)	−0.0062 (2)
P2	0.0529 (3)	0.0452 (3)	0.0409 (3)	0.0026 (2)	−0.0055 (2)	−0.0013 (2)
S2	0.0355 (2)	0.0422 (3)	0.0729 (3)	−0.0058 (2)	0.0009 (2)	0.0035 (2)
P1	0.0472 (3)	0.0532 (3)	0.0423 (3)	−0.0029 (2)	−0.0002 (2)	0.0065 (2)
O1	0.0383 (8)	0.0691 (11)	0.0856 (12)	0.0028 (7)	0.0084 (7)	0.0101 (9)
C7	0.0424 (11)	0.0387 (10)	0.0479 (10)	0.0035 (8)	0.0032 (8)	0.0037 (8)
C9	0.0377 (9)	0.0518 (11)	0.0442 (10)	0.0056 (8)	0.0000 (7)	0.0026 (9)
O2	0.0976 (14)	0.0380 (9)	0.1097 (15)	0.0031 (8)	−0.0022 (11)	−0.0105 (9)
C10	0.0428 (10)	0.0435 (10)	0.0358 (9)	0.0075 (8)	0.0015 (7)	−0.0015 (8)
N1	0.0616 (11)	0.0483 (10)	0.0458 (9)	0.0136 (8)	0.0018 (7)	−0.0040 (7)
C8	0.0510 (11)	0.0384 (11)	0.0613 (12)	0.0032 (8)	−0.0005 (9)	0.0009 (9)
C12	0.0460 (12)	0.0879 (19)	0.0661 (14)	0.0269 (13)	0.0043 (10)	0.0017 (13)
C11	0.0665 (14)	0.0675 (15)	0.0428 (10)	0.0308 (13)	0.0019 (9)	−0.0026 (10)
C6	0.0600 (13)	0.0693 (15)	0.0584 (12)	0.0014 (11)	−0.0192 (10)	0.0096 (11)
N2	0.0382 (9)	0.0730 (13)	0.0723 (12)	0.0083 (9)	0.0024 (8)	0.0048 (10)
C2	0.0597 (14)	0.135 (3)	0.0548 (13)	0.0022 (16)	−0.0134 (11)	0.0142 (15)
C3	0.152 (3)	0.081 (2)	0.0660 (16)	−0.043 (2)	−0.0189 (17)	0.0327 (15)
C4	0.0638 (14)	0.099 (2)	0.0552 (12)	−0.0068 (14)	0.0009 (11)	0.0253 (13)
C1	0.0862 (18)	0.112 (2)	0.0493 (13)	0.0191 (17)	0.0164 (12)	−0.0033 (14)
C5	0.160 (3)	0.076 (2)	0.0591 (15)	0.0244 (19)	−0.0146 (17)	−0.0217 (14)

### Geometric parameters (Å, °)

**Table:**

Fe1—C8	1.761 (2)	C12—C11	1.352 (4)
Fe1—C7	1.780 (2)	C12—H12A	0.9300
Fe1—P1	2.2793 (6)	C11—H11A	0.9300
Fe1—P2	2.2840 (6)	C6—H6A	0.9600
Fe1—S2	2.3058 (6)	C6—H6B	0.9600
Fe1—S1	2.3170 (6)	C6—H6C	0.9600
S1—C10	1.7488 (18)	C2—H2B	0.9600
P2—C5	1.811 (3)	C2—H2C	0.9600
P2—C6	1.812 (2)	C2—H2D	0.9600
P2—C4	1.817 (2)	C3—H3A	0.9600
S2—C9	1.745 (2)	C3—H3B	0.9600
P1—C1	1.808 (2)	C3—H3C	0.9600
P1—C2	1.809 (2)	C4—H4A	0.9600
P1—C3	1.810 (3)	C4—H4B	0.9600
O1—C7	1.134 (2)	C4—H4C	0.9600
C9—N2	1.332 (2)	C1—H1B	0.9600
C9—C10	1.418 (3)	C1—H1C	0.9600
O2—C8	1.142 (3)	C1—H1D	0.9600
C10—N1	1.330 (3)	C5—H5A	0.9600
N1—C11	1.343 (3)	C5—H5B	0.9600
C12—N2	1.349 (3)	C5—H5C	0.9600
			
C8—Fe1—C7	94.81 (9)	N1—C11—C12	122.6 (2)
C8—Fe1—P1	91.05 (7)	N1—C11—H11A	118.7
C7—Fe1—P1	90.32 (6)	C12—C11—H11A	118.7
C8—Fe1—P2	90.95 (7)	P2—C6—H6A	109.5
C7—Fe1—P2	91.08 (6)	P2—C6—H6B	109.5
P1—Fe1—P2	177.45 (2)	H6A—C6—H6B	109.5
C8—Fe1—S2	86.14 (6)	P2—C6—H6C	109.5
C7—Fe1—S2	176.78 (6)	H6A—C6—H6C	109.5
P1—Fe1—S2	86.58 (2)	H6B—C6—H6C	109.5
P2—Fe1—S2	91.98 (2)	C9—N2—C12	115.7 (2)
C8—Fe1—S1	174.39 (7)	P1—C2—H2B	109.5
C7—Fe1—S1	90.01 (6)	P1—C2—H2C	109.5
P1—Fe1—S1	91.79 (2)	H2B—C2—H2C	109.5
P2—Fe1—S1	86.09 (2)	P1—C2—H2D	109.5
S2—Fe1—S1	89.198 (19)	H2B—C2—H2D	109.5
C10—S1—Fe1	103.59 (7)	H2C—C2—H2D	109.5
C5—P2—C6	102.18 (13)	P1—C3—H3A	109.5
C5—P2—C4	102.93 (15)	P1—C3—H3B	109.5
C6—P2—C4	102.08 (12)	H3A—C3—H3B	109.5
C5—P2—Fe1	116.29 (10)	P1—C3—H3C	109.5
C6—P2—Fe1	116.96 (8)	H3A—C3—H3C	109.5
C4—P2—Fe1	114.31 (8)	H3B—C3—H3C	109.5
C9—S2—Fe1	103.89 (7)	P2—C4—H4A	109.5
C1—P1—C2	102.28 (13)	P2—C4—H4B	109.5
C1—P1—C3	103.18 (15)	H4A—C4—H4B	109.5
C2—P1—C3	103.19 (14)	P2—C4—H4C	109.5
C1—P1—Fe1	117.49 (9)	H4A—C4—H4C	109.5
C2—P1—Fe1	115.19 (8)	H4B—C4—H4C	109.5
C3—P1—Fe1	113.66 (9)	P1—C1—H1B	109.5
O1—C7—Fe1	178.51 (19)	P1—C1—H1C	109.5
N2—C9—C10	121.58 (19)	H1B—C1—H1C	109.5
N2—C9—S2	116.69 (17)	P1—C1—H1D	109.5
C10—C9—S2	121.72 (14)	H1B—C1—H1D	109.5
N1—C10—C9	121.13 (17)	H1C—C1—H1D	109.5
N1—C10—S1	117.51 (15)	P2—C5—H5A	109.5
C9—C10—S1	121.35 (14)	P2—C5—H5B	109.5
C10—N1—C11	116.28 (19)	H5A—C5—H5B	109.5
O2—C8—Fe1	176.9 (2)	P2—C5—H5C	109.5
N2—C12—C11	122.7 (2)	H5A—C5—H5C	109.5
N2—C12—H12A	118.7	H5B—C5—H5C	109.5
C11—C12—H12A	118.7		
			
C8—Fe1—S1—C10	−29.4 (8)	S2—Fe1—P1—C2	178.35 (12)
C7—Fe1—S1—C10	−178.74 (8)	S1—Fe1—P1—C2	89.26 (12)
P1—Fe1—S1—C10	90.93 (6)	C8—Fe1—P1—C3	23.20 (15)
P2—Fe1—S1—C10	−87.66 (6)	C7—Fe1—P1—C3	118.01 (15)
S2—Fe1—S1—C10	4.37 (6)	P2—Fe1—P1—C3	−118.6 (5)
C8—Fe1—P2—C5	6.40 (15)	S2—Fe1—P1—C3	−62.87 (13)
C7—Fe1—P2—C5	−88.43 (14)	S1—Fe1—P1—C3	−151.96 (13)
P1—Fe1—P2—C5	148.2 (5)	C8—Fe1—C7—O1	78 (8)
S2—Fe1—P2—C5	92.57 (13)	P1—Fe1—C7—O1	−13 (8)
S1—Fe1—P2—C5	−178.37 (13)	P2—Fe1—C7—O1	169 (8)
C8—Fe1—P2—C6	−114.62 (11)	S2—Fe1—C7—O1	−29 (9)
C7—Fe1—P2—C6	150.55 (11)	S1—Fe1—C7—O1	−105 (8)
P1—Fe1—P2—C6	27.1 (5)	Fe1—S2—C9—N2	−177.54 (14)
S2—Fe1—P2—C6	−28.45 (9)	Fe1—S2—C9—C10	1.34 (16)
S1—Fe1—P2—C6	60.61 (9)	N2—C9—C10—N1	2.8 (3)
C8—Fe1—P2—C4	126.20 (12)	S2—C9—C10—N1	−176.04 (14)
C7—Fe1—P2—C4	31.37 (12)	N2—C9—C10—S1	−178.48 (14)
P1—Fe1—P2—C4	−92.0 (5)	S2—C9—C10—S1	2.7 (2)
S2—Fe1—P2—C4	−147.63 (10)	Fe1—S1—C10—N1	173.69 (13)
S1—Fe1—P2—C4	−58.57 (10)	Fe1—S1—C10—C9	−5.10 (16)
C8—Fe1—S2—C9	173.53 (10)	C9—C10—N1—C11	−0.9 (3)
C7—Fe1—S2—C9	−79.2 (11)	S1—C10—N1—C11	−179.71 (13)
P1—Fe1—S2—C9	−95.18 (7)	C7—Fe1—C8—O2	153 (4)
P2—Fe1—S2—C9	82.72 (7)	P1—Fe1—C8—O2	−117 (4)
S1—Fe1—S2—C9	−3.34 (7)	P2—Fe1—C8—O2	62 (4)
C8—Fe1—P1—C1	143.77 (13)	S2—Fe1—C8—O2	−30 (4)
C7—Fe1—P1—C1	−121.41 (13)	S1—Fe1—C8—O2	4(5)
P2—Fe1—P1—C1	2.0 (5)	C10—N1—C11—C12	−1.2 (3)
S2—Fe1—P1—C1	57.70 (12)	N2—C12—C11—N1	1.8 (3)
S1—Fe1—P1—C1	−31.39 (12)	C10—C9—N2—C12	−2.2 (3)
C8—Fe1—P1—C2	−95.58 (14)	S2—C9—N2—C12	176.63 (16)
C7—Fe1—P1—C2	−0.76 (13)	C11—C12—N2—C9	0.1 (3)
P2—Fe1—P1—C2	122.7 (5)		
